# 2024 Acknowledgment of AAC Reviewers

**DOI:** 10.1128/aac.00334-25

**Published:** 2025-04-02

**Authors:** Cesar A. Arias

**Affiliations:** 1 Houston Methodist Hospital 23534 , Houston, Texas, USA; 2 Houston Methodist Research Institute 167626 , Houston, Texas, USA; 3 Weill Cornell Medical College 12295 , New York, New York, USA

## EDITORIAL

*Antimicrobial Agents and Chemotherapy*’s high quality and timely publication of groundbreaking scientific research are critically dependent on the outstanding efforts of the editorial board members (https://journals.asm.org/journal/aac/board-editors), who generously contribute their time, wisdom, and talents to our journal. The editors of AAC also acknowledge the *ad hoc* referees who have contributed their reviews in the past year, and we express our deep appreciation for their contributions to maintaining the journal’s scientific rigor, relevance of research, and high clinical impact.

Lilian M. Abbo

Mohd Hafiz Abdul-Aziz

Craig Aboltins

Robert B. Abramovitch

Amanda N.D. Adams

Paul C. Adamson

Max W. Adelman

Amos Adler

Prakhar Agrawal

Sarah A. Ahmed

Yukihiro Akeda

Alexandre Alanio

Mahableshwar Shankar Albur

Kevin Alby

Katie J. Aldred

Courtney Aldrich

Jan-Willem Alffenaar

Innocent Ali

George P. Allen

Kyle R. Allison

Abdulaziz S. Alobaid

Sara Alosaimy

J. Andrew Alspaugh

Abdullah Alsultan

Deena R. Altman

Alfred Amambua-Ngwa

Paul G. Ambrose

Nicole C. Ammerman

Xinwei An

Tessa Andermann

Deborah M. Anderson

Peter Anderson

David R. Andes

Jacopo Angelini

Richard M. Anthony

Alberto Antonelli

Andres Aranda-Diaz

Jesús Aranda

Jorge Arca-Suárez

Maiken Cavling Arendrup

Robert Ariano

Corinne Arpin

Antonio Arrieta

Ruth Ashbee

Usama Ashraf

Saima Aslam

Ina Attrée

Yoann Augagneur

Saranya Auparakkitanon

Julie Autmizguine

Yossef Av-Gay

Sean N. Avedissian

Matthew B. Avison

Malik Aydin

Francine J. Azeredo

Ahmed Babiker

Joanna Bacon

Hamid Badali

Aranya Bagchi

Anthony Bai

Sarah L. Baines

Sheridan J.C. Baker

Fernando Baquero

Beatriz Baragana

Vito Baraka

Galileu Barbosa Costa

Francesco Barchiesi

Daniel Barkan

Raul G. Barletta

Imke Heleen Bartelink

Natália Barth

Robert H. Bates

Thomas Bauch

Sarah R. Beattie

Alejandro Beceiro

Zhu-Chun Bei

Romuald Bellmann

Gustavo Benaim

Thomas Benfield

Jose A. Bengoechea

Andrej Benjak

Youssef Bennis

Elizabeth L. Berkow

Judith Berman

Jesús Bermejo-Martin

Luiz E. Bermudez

Joseph S. Bertino

Julie Bertrand

Asish K. Bhattacharya

Debapriya Bhattacharya

Souvik Bhattacharyya

Adarsh Bhimraj

Hongkai Bi

Ulrike Binder

Lyn-Marie Birkholtz

Todd Black

Patrick J. Blackall

Lucia Blasco

Joseph M. Blondeau

Lucas Boeck

Balazs Bogos

Joseph M. Boll

Remy A. Bonnin

Robert A. Bonomo

P. Brandon Bookstaver

Aleksandra Borek

Margarida Borges

Jeffrey L. Bose

Joao Botelho

Dennis Botman

Germán Bou

Naim Bouazza

Cara C. Boutte

Thomas Briese

Adrian Brink

Shaun R. Brinsmade

Rachel S. Britt

Joanna S. Brooke

Roland Brosch

Isabel Brosius

Eric D. Brown

James C.M. Brust

Alexandra L. Bryson

Shilpa Buch

Whitney R. Buckel

Nienke Buddelmeijer

Jochem Berend Buil

Jürgen B. Bulitta

Charles Burdet

David Burger

James Burns

Vincent Burrus

Ignacio Cabarello

Richard A. Calderone

Dante Calise

Emmanuelle Cambau

David R. Cameron

Stephane Canaan

Bruno Canard

Edmund V. Capparelli

Glen P. Carter

James E. Cassat

Juan Alberto Castillo-Garit

Dario Cattaneo

Igor Cestari

Sun-Shin Cha

Apisit Chaidee

Kitti Wing Ki Chan

Xavier Charpentier

Som S. Chatterjee

Vishnu Chaturvedi

Marina Chavchich

Dingqiang Chen

Jiazhen Chen

Qingquan Chen

Qiong Chen

Xinchun Chen

Yi-Chih Chen

Yunyu Chen

Tong Cheng

Melissa D. Chengalroyen

Jerald Cherian

Chuan Chiang-Ni

Laurent Roberto Chiarelli

Kelly Chibale

Emmanuel Chigutsa

Kathleen Chiotos

Chien-Shun Chiou

Ute Chiriac

Andrew Chou

Haiqing Chu

Tom Coenye

Dina Coertzen

Stuart H. Cohen

Pier Giorgio Cojutti

Joseph M. Colacino

David C. Coleman

James Collins

Benjamin Colton

Fabrice Compain

Chris Connor

Paul J. Converse

Laura C. Cook

Brendan Cormack

Nicolas W. Cortes-Penfield

Christopher Cousins

Leah E. Cowen

Frédéric Crémazy

Allister Crow

Liwang Cui

Richard D. Cummings

Christina A. Cuomo

Scott R. Curry

Jessica Cusato

Gabriela Jorge Da Silva

Silvia Amaral Gonçalves Da Silva

Kurt Martin Dahlstrom

Christopher Darlow

Rachel Louise Darnell

Raymond James Dattwyler

Michael Z. David

Bryan Davies

Anne Veronique Davin-Regli

Margaret A. Davis

Paul H. Davis

Carolina de Miranda Silva

Rodrigo de Paula Baptista

Yannick Debing

Anjan Debnath

Mary Ann DeGroote

Sarah Dellière

Vincent Delorme

Thomas G. Denes

Xufang Deng

Paolo Denti

Keith M. Derbyshire

Aaron Sriram Devanathan

Gunaraj Dhungana

Jose A. Di Conza

Y. Peter Di

Andreas H. Diacon

Mahamadou Diakite

Mederic Diard

Lorena Diaz

Joseph P. Dillard

Jo-Anne R. Dillon

Nicholas A. Dillon

Tanis C. Dingle

Genevieve Shirley Dobihal

Jean-Denis Docquier

Elizabeth Dodds Ashley

Yalin Dong

Curtis Donskey

Kelly E. Dooley

Abby Douglas

Kevin J. Downes

Charles M. Dozois

Erwin Dreesen

Dimitri Drekonja

Philip G. Drennan

Djamel Drider

George L. Drusano

J.P. Dubey

Sandra Duffy

Lisa E. Dumkow

Julie B. Dumond

Michael W. Dunne

Julie C. Dunning Hotopp

Sheetij Dutta

Jeffrey D. Dvorin

Kathryn Dzintars

Michael D. Edstein

Stephanie Lynn Egge

Amer El Ghali

Christopher A. Elkins

Jose Antonio Escudero

German Esparza

Socorro Espuelas

Valeria Fabre

Marco Falcone

Sven Falke

Majid Fasihi Harandi

Sizhou Feng

Miguel Fernández-Huerta

Aude A. Ferran

Kia Ferrell

Scott G. Filler

Jacqueline Findlay

Suzanne M.J. Fleiszig

Derek Fleming

Steven L. Foley

Juan Fontana

Jarrod R. Fortwendel

Simon J. Foster

Muinah Adenike Fowora

Bénédicte Franck

Evelyn J. Franco

Patrice Francois

Dara W. Frank

Kristi L. Frank

Scott G. Franzblau

Ana R. Freitas

Jean Marie A. Frere

Niels Frimodt Möller

Jonathan Gray Frye

Frieder Fuchs

Bożena Futoma-Kołoch

Fajri Gafar

Moreno Galleni

Qian Gao

Scott Gary Franzblau

Ulises Garza-Ramos

David C. Gaston

Yuqing Geng

Martin Gengenbacher

Don Bambino Geno Tai

Pallavi Ghosh

Daniele Roberto Giacobbe

Tommaso Giani

Brigitte Gicquel

Stacey D. Gilk

Christian M. Gill

Mathieu Gissot

Stefano Giulieri

Alicia Gomez-Lopez

Angela Gomez-Simmonds

Sonia Alejandra Gomez

Marta Gontijo Aguiar

Lisandro J. González

Kellie J. Goodlet

Christopher Dean Goodman

Sylvain Goutelle

Stephen Graves

Todd A. Gray

David E. Greenberg

David C. Griffith

David E. Griffith

Katarzyna Grzyb

Shuang-Xi Gu

Paul Gubbins

François Guérin

Jesus Guinea

Hannah Gunter

Ju-Tao Guo

Kalpana Gupta

Shashank Gupta

Félix Gutiérrez

Maria Hadjifrangiskou

Ruth M. Hall

Otavio Hallal Ferreira Raro

Robert Hammond

Christopher Harmer

Patrick N.A. Harris

Lee H. Harrison

Kelley R. Healey

Peter Heisig

Georg Hempel

Andrew Henderson

Isabelle G. Henshall

Morgan R. Herod

Paul G. Higgins

Eberhard Hildt

B. Joseph Hinnebusch

Maya Christina Hites

Michael R. Hodges

Martin Hoenigl

Emily B. Hollister

Kara S. Hood

David C. Hooper

William W. Hope

Yasuhiro Horita

Jim-Tong Horng

Peter Hortschansky

Angela Houston

Jessica R. Howard-Anderson

Jaroslav Hrabak

Fupin Hu

Xiaoting Hua

Fenglei Huang

Si-Yang Huang

Vanthida Huang

Yanqin Huang

Johannes Huebner

Romney M. Humphries

Anna R. Huppler

Ashraf S. Ibrahim

Elisa H. Ignatius

Natalia A. Ilyushina

Francesco Imperi

Dilek Ince

Timothy J.J. Inglis

Bogdan I. Iorga

Deus S. Ishengoma

Nabila Ismail

Brent Iverson

Masaharu Iwasaki

Cedric Jacqueline

Mahboob Jafari

Kim Janda

Jichan Jang

Nerea Jauregizar

Babak Javid

Katy Jeannot

Andrew Jezewski

Bijay Kumar Jha

Byung Woo Jhun

Haiyong Jia

Jhih-Hang Jiang

Yuefei Jin

Kyung-Wook Jo

Melissa D. Johnson

Regina Joice Cordy

Lorraine Jones-Brando

Lucy C. Jones

Siv Jönsson

Li Jun

Barbara C. Kahl

Manjula Kalia

Deepika Kannan

Daniel J. Kao

James A. Karlowsky

Mats O. Karlsson

Fatah Kashanchi

Hangjun Ke

Po-Yuan Ke

Razieh Kebriaei

Megan L. Kempher

Caroline Keroack

Ayesha Khan

Nina Khanna

Anupama Khare

Farnaz Kheirandish

Amal Khudair Khalaf

Daniel Muthui Kiboi

Nicolas Kieffer

John H. Kimbrough

Patrick Kinn

James E. Kirby

Frank Kloprogge

Wen-Chien Ko

Shinji Kobuchi

Clemens Kocken

Julia R. Köhler

Thilo Köhler

Milena Kordalewska

Izabela Korona-Glowniak

Libor Krasny

Andrea Kreidenweiss

Barry N. Kreiswirth

Laurent Kremer

Ruhul Kuddus

Wesley D. Kufel

Johanna Kuhlin

Nakwon Kwak

Maria-Halima Laaberki

Nico Lachmann

Maria Lafuente-Monasterio

Rahmi Lale

Gordon Langsley

Michael Lanzer

Jean-Philippe Lavigne

Quentin Le Hingrat

Christophe Le Terrier

Shuai Le

Thuy Le

Nathan A. Ledeboer

Chia Y. Lee

Francesca Lee

Jayhun Lee

Jean Y.H. Lee

Philippe Lehours

Jonathan Lellouche

Andrey Viktorovich Letarov

Jizhou Li

Ruichao Li

Shiyu Li

Pharath Lim

Gang Lin

Michael Lin

Xiaorong Lin

Yu-Wei Lin

Jamin Liu

Xiaolin Liu

Catherine Llanes

Catherine Loc-Carillo

Giovanni Longo

Céline Loot

Francisca Lopes

Thomas Louie

Kuan-Yi Lu

Brigid Lucey

Ju Luo

Bruna Lustri

Haifa Lyster

Liang Ma

Alasdair P. MacGowan

Karsten Maeder

Sophie Magreault

Tridib Mahata

Hossein Mahmoudvand

Thomas Maitre

Olga Makarova

Hedi Mammeri

Pooja Manchandani

Ujjini Manjunatha

Alexander Mankin

Rumyana Markovska

Claudia N.H. Marques

Luise Martin

Richard J. Martin

Erica dos Santos Martins-Duarte

Delphine Martiny

Anne-Grete Märtson

Mulaka Maruthi

Catia Marzolini

Elysia A. Masters

Kerrie L. May

Erin K. McCreary

Patrick McGann

David McGee

Edward E. McKee

Robert McLaughlin

Sarah M. McLeod

Case McNamara

Khisimuzi Mdluli

Eman Meabed

Krina Mehta

Monica Mehta

Rodrigo E. Mendes

Beatrice Mercorelli

Tuuli Metsvaht

Rosa del Carmen Milán-Segovia

Alita A. Miller

Eric J. Miller

John Misasi

Satoshi Mitarai

Aaron P. Mitchell

Greg Moeck

Ousman Mohammed

Maria F. Mojica

Sachel S. Mok

Robert E. Molestina

Virginie Molle

Rubens Monte Neto

Lianet Monzote

Atica Moosa

Muhammed S. Moosa

Yuji Morita

Tatum D. Mortimer

Lawrence I. Mortin

Michael Mourez

Samir H. Moussa

Bartolome Moya

Anouk E. Muller

Erik Munson

Mark Murphy

Daniel M. Musher

Joe S. Mymryk

Alo Nag

Esteban C. Nannini

Navaneeth Narayanan

Jerry Nedelman

Brian C. Nelson

Catherine Neuwirth

Caroline L. Ng

Paul Nguewa

Tran-Bach Nguyen

Melanie R. Nicol

David P. Nicolau

Angela M. Nilius

Camus Nimmo

Tomoyasu Nishimura

David E. Nix

Patrice Nordmann

Ângela Novais

Christian Nsanzabana

Eric L. Nuermberger

Mariia Numi

Maria Nuñez

Dennis Nurjadi

Myat Htut Nyunt

Teresa R. O'Meara

B. Obura

Ifedayo Victor Ogungbe

Anil Ojha

Kayode K. Ojo

Alessandra Oliva

Jolien Onsea

Timothy J. Opperman

Mehmet A. Orman

Joshua Osowicki

Puey Ounjai

Dafydd Owen

Joel S. Owen

Timothy Palzkill

Cihan Papan

Sunil Parikh

Tanya Parish

Nicole M. Parrish

Alexander O. Pasternak

David Paterson

Federico Pea

Melanie M. Pearson

Charles A. Peloquin

José Penades

Junping Peng

Federico Perez

David S. Perlin

Jody Phelan

Stephane Picot

Jean-Paul Pirnay

Sven Pischke

Daniel Pletzer

Mateusz Plucinski

Tzvi Pollock

Husain Poonawala

Spyros Pournaras

Michael Povelones

Erica Prochaska

Daofeng Qu

Yok-Ai Que

Helena Rabie

Meena Ramchandani

Michael Ramharter

Maria Soledad Ramirez

Santiago Ramon-Garcia

Amit Ranjan

Gauri G. Rao

Magnus Rasmussen

Michael D. Reed

Xiaomei Ren

Adrian Richter

Thomas V. Riley

Martijn Riool

Michael K. Riscoe

Octavio Miguel Rivero-Lezcano

Nicole Robbins

Scott C. Roberts

Gregory T. Robertson

Sara M. Robledo

Jeremy Rock

Ana Paula Drummond Rodrigues

Jose-Manuel Rodriguez-Martinez

Juan Carlos Rodriguez

Mario Alberto Rodríguez

Paul D. Roepe

David Rogers

Kyle H. Rohde

Jesus Rojas-Jaimes

R. Martin Roop

Antoine Roquilly

Gus R. Rosania

Gian Maria Rossolini

Joaquin Rullas-Trincado

Etienne Ruppé

Jeffrey Ryback

Michael J. Rybak

Mustafa Sadek

Orhan Sahin

Jumpei Saito

Makoto Saito

Max Salfinger

Sam Salman

Michael Joseph Satlin

Toyotaka Sato

Tor Savidge

Joy Scaria

Kimberly Scarsi

Marc H. Scheetz

Thomas Scheier

Patrick M. Schlievert

Thomas Schön

Audrey N. Schuetz

Michael J. Schurr

Martin Schutten

Effie Scoulica

Salome N. Seiffert

Philip R. Selby

Eric Senneville

Alisa W. Serio

Yechiel Shai

Dennis Shanks

Sima Sharara

Amit Sharma

Umender Sharma

Justin Shiau

Thomas M. Shinnick

William C. Shropshire

Faiza A. Siddiqui

Tânia Silva

Patricia J. Simner

Dhiraj Kumar Singh

Sanjay Singh

Vinayak Singh

Marion J. Skalweit

Andrew Skinner

Derek James Sloan

Andrea María Smania

Kenneth P. Smith

David R. Snydman

Olusegun O. Soge

Nicolas Soler

Vijay Soni

Fritz Sörgel

Dolors Soy

Owen B. Spiller

Stanley M. Spinola

Divya Sriram

Anand Srivastava

Joseph F. Standing

Joerg Steinmann

Brian Stevenson

Peter J. Stogios

D.G. Storey

Ana Streling de Oliveira

Frank Stubenrauch

Wenji Su

Xin-zhuan Su

David Sue

Philip Noel Suffys

Bao Sun

Jianjun Sun

Sesh A. Sundararaman

Arnfinn Sundsfjord

Heungsup Sung

Masahiro Suzuki

Din Syafruddin

Melissa Louise Sykes

Yi-Wei Tang

Carlo Tascini

Tobias Tenenbaum

Nobuyuki Tetsika

Joshua T. Thaden

George Thompson

Michael Thy

Leann Tilley

Jean-François Timsit

Boghuma K. Titanji

Steven Y. C. Tong

Gabriela Torrea

Antoni Torres

Sultan Tousif

D.J. Touw

Barbara W. Trautner

Jai Justin Tree

Tamara L. Trienski

Renée M. Tsolis

Adrianna M. Turner

Nicholas A. Turner

Frank Tverdek

Carles Ubeda

Tsuyoshi Uehara

Wanchana Ungphakorn

Julio A. Urbina

Françoise Van Bambeke

Bram Van den Bergh

Nicole N. van der Wel

H. Rogier van Doorn

Sebastiaan J. van Hal

Daria Van Tyne

Wesley C. Van Voorhis

Brian C. VanderVen

Brian D. VanScoy

Jose Vargas-Muniz

Mandira Varma-Basil

Shawn Vasoo

Roberto Vázquez

Luis Alberto Vega

Tony Velkov

Carola Venturini

Omar Vesga

Nicolas Veziry

J. Alexander Viehman

Rafael Vignoli

Miguel Viveiros

Isabelle Vock

Crista B. Wadsworth

Jennifer N. Walker

Pin Wan

Yu Wan

Hongliang Wang

Minggui Wang

Shih-Min Wang

Robert Ward

Matthew Wargo

Robin M. Warren

Suzanne Louise Warring

Roeland E. Wasmann

Heather Weerdenburg

Wei Wei

Yahan Wei

David Weiss

Melanie Wellington

Janett Wennek-Klose

Guido Werner

Neeva Wernsman Young

Brian James Werth

David Wesche

Bryan P. White

Michael Gordon Whitfield

Sebastian G. Wicha

Julia Willett

Francis Williams Ojara

Noelle Williams

Riley J. Williams

Danny W. Wilson

Nikolai Winbichler

Ian Windham

Marisa Winkler

Benoit Witkowski

Zenaw Wollie

William R. Wolowich

Tania Wong Fok Lung

Aniela Wozniak

Rachel A.F. Wozniak

Hung-Jen Wu

Linxuan Wu

Juliane Wunderlich

Yan Xiang

Li Xiao

Stanley C. Xie

Sifei Xing

Yoshiharu Yamaichi

Keizo Yamamoto

Song Yang

Yang Yang

Rebecca Yee

Fei Yu

Xuben Yu

Yunsong Yu

Jing Yuan

Shuofeng Yuan

Vyacheslav Yurchenko

Raffaele Zarrilli

Merve S. Zeden

Anne-Marie Zeeman

Markus Zeitlinger

Sheryl Zelenitsky

Min Zeng

Jonathan Zenilman

Bin Zhang

Huimin Zhang

Jing Zhang

Qijing Zhang

Shuye Zhang

Wei Zhang

Hanjun Zhao

Qin Zhao

Wei Zhao

Datong Zheng

Tieli Zhou

Xiaohui Zhou

Zhemin Zhou

Duolong Zhu

Xiao Zhu

Jian-Ping Zuo

